# Extracellular Matrix Proteins Expression Profiling in Chemoresistant Variants of the A2780 Ovarian Cancer Cell Line

**DOI:** 10.1155/2014/365867

**Published:** 2014-04-03

**Authors:** Radosław Januchowski, Piotr Zawierucha, Marcin Ruciński, Michał Nowicki, Maciej Zabel

**Affiliations:** ^1^Department of Histology and Embryology, Poznan University of Medical Sciences, Poland Święcickiego 6 Street, 61-781 Poznan, Poland; ^2^Departament of Anatomy, Poznan University of Medical Sciences, Poland Święcickiego 6 Street, 61-781 Poznan, Poland; ^3^Department of Histology and Embryology, Wroclaw Medical University, Poland Chałubińskiego 6A Street, 50-368 Wroclaw, Poland

## Abstract

Ovarian cancer is the leading cause of death among gynaecological malignancies. Extracellular matrix (ECM) can affect drug resistance by preventing the penetration of the drug into cancer cells and increased resistance to apoptosis. This study demonstrates alterations in the expression levels of ECM components and related genes in cisplatin-, doxorubicin-, topotecan-, and paclitaxel-resistant variants of the A2780 ovarian cancer cell line. Affymetrix Gene Chip Human Genome Array Strips were used for hybridisations. The genes that had altered expression levels in drug-resistant sublines were selected and filtered by scatter plots. The genes that were up- or downregulated more than fivefold were selected and listed. Among the investigated genes, 28 genes were upregulated, 10 genes were downregulated, and two genes were down- or upregulated depending on the cell line. Between upregulated genes 12 were upregulated very significantly—over 20-fold. These genes included COL1A2, COL12A1, COL21A1, LOX, TGFBI, LAMB1, EFEMP1, GPC3, SDC2, MGP, MMP3, and TIMP3. Four genes were very significantly downregulated: COL11A1, LAMA2, GPC6, and LUM. The expression profiles of investigated genes provide a preliminary insight into the relationship between drug resistance and the expression of ECM components. Identifying correlations between investigated genes and drug resistance will require further analysis.

## 1. Introduction

Among gynaecologic malignancies ovarian cancer is the leading cause of deaths. The average 5-year survival is approximately 40%, but patients with advanced disease (stages III and IV according to FIGO classification) have a significantly lower survival rate of only 10–20% [1]. High mortality among ovarian cancer patients results from late diagnosis and low effectiveness of chemotherapy. Regardless of the stage of the disease, the first line of chemotherapy consists of a combined chemotherapeutic regimen of platinum and taxane [2]. The second line of treatment usually includes taxane, cisplatin (Cis), topotecan (Top), and doxorubicin (Dox) [3, 4].

The main reason of low chemotherapy effectiveness is drug resistance of cancer cells. Cellular mechanisms of drug resistance are various. They include lower accumulation of the drug in the cells, change in localization of the drug in the cell, slower inactivation of the drug, faster repair of damage by the drug DNA and cellular membranes as well as increased ability for tolerance of these damages, and changes in molecular targets, which make them insensitive or less sensitive to the drugs' actions, changes in gene expression, and changes in regulation of apoptosis. However, the most significant and frequently occurring mechanism of drug resistance is multiple drug resistance (MDR). It means the ability of cancer cells to actively remove drugs from the cell via transport proteins. The most important proteins taking part in this process are proteins belonging to ABC family, and among them the best known is glycoprotein P [5].

Although we know a lot about chemotherapy resistance, sometimes it is difficult to explain response of cancer cells to cytostatic drugs on the basis of expression profile of genes involved in drug resistance. This indicates that other unknown yet genes can also participate in cancer drug resistance. Genome wide expression analysis by oligonucleotide microarray is a powerful molecular tool for the discovery of new genes involved in molecular processes including drug resistance.

Extracellular matrix (ECM) is made up of ground substance and fibers. The ground substance consists of proteoglycans like syndecan and lumican, multiadhesive glycoproteins like fibronectin and laminin, and glycosaminoglycans. Between fibers we can distinguish between collagen and elastin fibers. These molecules control many aspects of cell life such as gene expression, cell proliferation, differentiation, migration, adhesion, and cancer metastasis [6]. Expression of ECM proteins is limited to connective tissue in physiological condition and however has also been reported in many cancers* in vivo* [7] and in drug-resistant cancer cell lines [8].

It has been reported that ECM can affect drug resistance by preventing the penetration of the drug in the cancer cell [9–11]. Using different anticancer drugs researchers showed lower penetration of drugs through multicellular layer of cancer cells expressing laminins and collagens [10]. Interaction between cancer cells and interaction with components of ECM and with grow factors can affect the apoptosis sensitivity and drug resistance of cancer cells [12, 13]. This phenomenon is designated as a cell adhesion mediated drug resistance (CAM-DR) [14]. It has been reported that some tumours can develop drug resistance* in vivo* but not* in vitro*. This can be related to tumour microenvironment and expression of ECM [15]. Some researchers even believe that tumour microenvironment is a dominant force in drug resistance [16]. Changes in expression of ECM proteins, matrix metalloproteinases (MMPs), and other enzymes can lead to remodelling of ECM and increase cancer metastasis [17, 18].

This study shows alterations in the gene expression levels of ECM proteins in the Cis-resistant (A2780CR1, A2780CR2), paclitaxel (Pac)-resistant (A2780PR1, A2780PR2), Dox-resistant (A2780DR1, A2780DR2), and Top-resistant (A2780TR1, A2780TR2) variants of the A2780 ovarian cancer cell line.

## 2. Material and Methods

### 2.1. Cell Lines and Cell Culture

#### 2.1.1. The Human Ovarian Carcinoma Cell Line A2780 Was Purchased from ATCC

A2780 sublines that were resistant to Cis (A2780CR1, A2780CR2), Pac (A2780PR1, A2780PR2), Dox (A2780DR1, A2780DR2), and Top (W1TR1, W1TR2) were generated by the exposure of the A2780 cell line to incremental increases in the concentrations of the relevant drugs. The final concentrations of each drug were 1000 ng/mL Cis, 1100 ng/mL Pac, 100 ng/mL Dox, and 24 ng/mL Top. These concentrations were based on the work of Dietel et al. in 1993 [19] and were twofold greater than the plasma concentrations of the respective drugs 2 hours after intravenous administration. All the cell lines were maintained as monolayers in complete medium (MEM medium supplemented with 10% (*v*/*v*) foetal bovine serum, 2 pM L-glutamine, penicillin (100 units/mL), streptomycin (100 units/mL), and amphotericin B (25 *μ*g/mL)) at 37°C in a 5% CO_2_ atmosphere.

#### 2.1.2. RNA Isolation, cDNA Synthesis, and Target Preparation

RNA was isolated from A2780 and all the resistant sublines using the TRI Reagent (Sigma, St Louis, MO, USA), according to the manufacturer's instructions. The absorbance values (260, 280 nm) were measured for RNA quantification by spectrophotometry. The intactness of the extracted RNA was checked by electrophoresis using a 1% *w*/*v* denaturing agarose gel. Additionally, all samples were checked on a Bioanalyser 2100 (Agilent Technologies, Inc., Santa Clara, CA, USA). The evaluated RIN was between 8.5 and 10, with average of 9.2. Each RNA sample was diluted to 100 ng/*μ*L with an OD260/OD280 ratio of 1.8–2.0. All RNA samples were prepared in triplicate. cDNA was synthesised in two steps (first strand synthesis and second strand synthesis) using the Affymetrix GeneChip 3′IVT Express Kit (Affymetrix, Santa Clara, CA, USA), according to the manufacturer's instructions. Biotin-labelled cRNA synthesis (IVT Labelling) and cRNA fragmentation were performed using the Affymetrix GeneChip Kit reagents, according to the procedure described in the Affymetrix GeneAtlas 3′IVT Express Kit technical manual.

### 2.2. Target Hybridisation and Scanning

Biotin-labelled and fragmented target cRNA samples were loaded into Affymetrix GeneChip (Human Genome U219) Array Strips together with controls cRNAs and oligo B2. The hybridisation procedure was conducted at 45°C for 16 h in an AccuBlock Digital Dry Bath (Labnet international, Inc.) hybridisation oven. The washing and staining procedure was performed using an Affymetrix GeneAtlas Fuidics Station according to the instructions in the technical manual. An Affymetrix GeneAtlas Imaging Station was used for scanning the arrays.

### 2.3. Data Analysis and Preparation of Gene Lists

**Figure 1:**
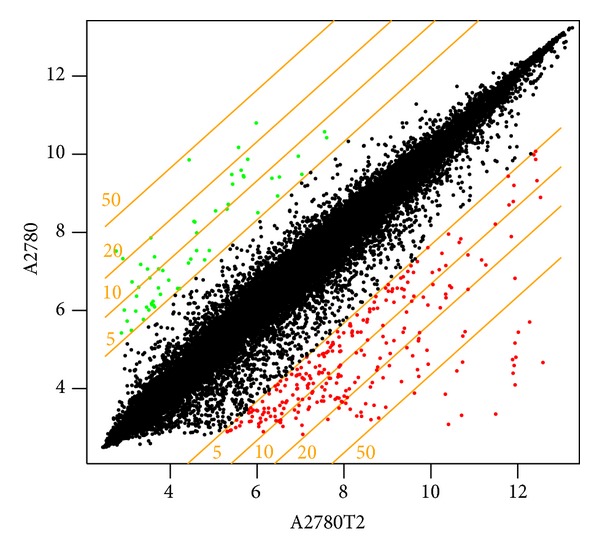
Scatter plot showing the fivefold up- and downregulated genes (the green and red dots, resp.) in the A2780TR2 cell line with respect to the Top-sensitive A2780 cell line. The plot filters the genes with fold-change values between 0.2- and 5-fold (black dots).

The preliminary analysis of the scanned chips was performed using Affymetrix GeneAtlas Operating software. The quality of gene expression data was checked according to quality control criteria provided by the software. Partek Express software (Partek, Inc., Chesterfield, MO, USA) was used for further data analysis and evaluation. Using quality control checkpoints and statistical analysis of gene fold-change significances, a table of the most important changes in gene expression was constructed. Next, the generated table was imported to Pathway Studio Explore (Ariadne Genomics, Rockville, MD, USA) where proper statistical analyses were carried out. To evaluate the *P* value indicating the significance of the enrichment score, a nonparametric statistical Mann-Whitney *U*-test was used (*α* = 0.05). Genes whose expression was significantly different between the resistant sublines and the parental A2780 cell line were listed. The upregulated and downregulated genes were selected. The genes were filtrated by scatter plot (Figure 1) and the genes that were upregulated and downregulated more than fivefold were considered when preparing gene lists. Finally, the genes that encode proteins related to ECM were selected from the gene list and a new list was generated to evaluate the relationship between these proteins and the drug-resistant phenotype.

## 3. Results

### 3.1. Gene Chip Scanning and Preliminary Analysis

The quality of all GeneChip expression data were in “good sample” limits according to preliminary data analysis parameters such as background and noise averages, percentage of present calls, presence of internal hybridization controls in increasing signals, presence of poly-A controls as decreasing signals, and GAPDH to beta actin 3′/5′ signal ratios.

### 3.2. Data Analysis, Gene Lists, and Evaluation

The genes that are related to ECM structure and metastatic property of cancers were selected. Analysis of these gene expressions in eight drug-resistant ovarian cancer cell lines can give some information about response of cancer cells to different cytostatic drugs treatment. Tables 1 and 2 summarize the alterations in ECM, matrix metallopeptidases, and related genes expression levels in drug-resistant sublines with respect to A2780 drug sensitive cell line. Significant changes greater than 5-fold and less than 0.2-fold (up-/downregulation over/below 5 and −5, resp.) were considered for evaluation of their contribution to drug resistance. The genes whose expression level changed in between 5- and 0.2-fold alteration were considered “not significant (NS)” in constructing gene lists.

Collectively, expression of 40 genes encoding ECM proteins, integrin receptors, matrix metallopeptidases, and related genes was changed in drug-resistant cell lines. 28 genes were upregulated in at least one drug-resistant cell lines. 10 genes were downregulated in at least one drug-resistant cell line and two genes were down- or upregulated dependent on the cell line. The most variable cell lines were one of the Pac-resistant cell line A2780PR1 and one of the Top-resistant cell line A2780TR2. In these cell lines we observed changes in 19 and 14 gene expression, respectively. The most stable cell line was Dox-resistant cell line A2780DR2; only three genes were upregulated.

Six genes were upregulated in both Top-resistant cell lines (A2780TR1, A2780TR2): ITGB8, COL1A2, TGFBI, LAMA4, HAPLN1, and MGP. One gene COL11A1 was downregulated in both Top-resistant cell lines. One gene LAMB1 was upregulated in both Pac-resistant cell lines (A2780PR1, A2780PR2). Three genes COL11A1, FN1, and KERA were downregulated in both Pac-resistant cell lines. One gene TIMP3 was upregulated and three genes COL11A1, FBN1, and KERA were downregulated in both Cis-resistant cell lines (A2780CR1, A2780CR2). Only one gene EPYC was upregulated in both Dox-resistant cell lines (A2780DR1, A2780DR2).

**Figure 2:**
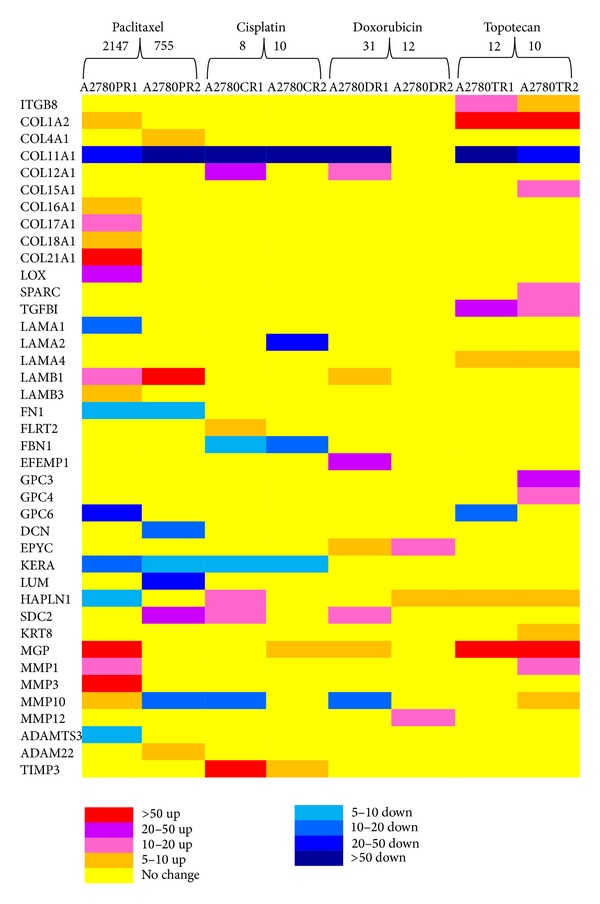
The expression ratios of ECM, matrix metallopeptidases, and related genes in the drug-resistant sublines. The numbers above cell lines indicate final resistance levels.

From 40 analysed genes, expression of 12 was upregulated very significantly—over 20-fold increase. These genes included COL1A2, COL12A1, COL21A1, LOX, TGFBI, LAMB1, EFEMP1, GPC3, SDC2, MGP, MMP3, and TIMP3. Four genes were very significantly downregulated: COL11A1, LAMA2, GPC6, and LUM. Changes in expression levels of all genes are summarised in Tables 1 and 2 and Figure 2.

## 4. Discussion 

This paper presents expression of genes encoding ECM and related proteins in eight drug-resistant ovarian cancer cell lines. The genes with fold-change values between 5 and 0.2 were considered to not be altered significantly, and the relationships between these genes and drug resistance will not be discussed.

The MDR phenotype of cancer cells is mainly related to expression of drug transporters from ABC family and among them the main players are glycoprotein P (P-gp) and breast cancer resistant protein (BCRP) [5]. Expression of these two genes in investigated cell lines was confirmed by qPCR (data not shown). Additionally, western blot analysis of BCRP protein was also correlated with the alterations in expression levels of the gene encoding this protein (data not shown).

However, increasing body of evidences indicates that tumour microenvironment [16] and expression of ECM proteins [7, 10, 11] can also play a very important role in tumour drug resistance. ECM may contribute to the drug resistance of solid tumours by preventing the penetration of therapeutic agents. Expression of ECM and related proteins is observed not only* in vivo* but also* in vitro* in drug resistance cell lines [8]. If cancer cells express ECM molecules together with transporters from ABC family* in vitro,* these molecules are likely to be important in drug resistance of cancer cells.

We observed upregulation of five collagen genes: COL1A2, COL16A1, COL17A1, COL18A1, and COL21A1 in A2780PR1 cell lines. In contrast only one collagen gene COL4A1 was upregulated in the A2780PR2 cell line. Thus expression of these genes seems not to be induced specifically by Pac but rather is one of the possibilities of cell response to this drug treatment. Similarly expression of COL12A1 was observed only in one of Cis- and one of Dox-resistant cell lines but not in the others. Very high expression level of COL1A2 in both Top-resistant cell lines suggests that expression of this collagen can be a specific cellular response to Top treatment. Taken together overexpression of at least one collagen gene was observed in six from eight drug-resistant cell lines. In four from these six cell lines expression was very high—over 20-fold increase. It has been observed by Netti et al. that more penetration-resistant tumours have extended collagen network [11]. Dense and tortuous tumour extracellular matrix can be a major barrier for drug delivery [20]. Diffusion rates for larger molecules inversely correlate with fibrillar collagen level, organization, and orientation [11, 21–23]. Expression of COL genes has also been observed by others in drug-resistant breast [8] and ovarian cancer cell lines [24]. In breast cancer cell line MCF-7 resistant to Vincristine (Vin), Pac, Docetaxel (Doc) and Dox, authors observed overexpression of six COL genes with the most abundant expression of COL4A1. In ovarian cancer cell lines resistant to Cis, overexpression of COL6A3 has been observed. Furthermore cultivation of Cis sensitive cells in the presence of collagen VI protein promoted resistance to Cis* in vitro. *This effect can result from interaction of collagen with cellular receptors leading to increased resistance to apoptosis [24]. One more possibility is that collagens specifically affect cytostatic drugs decreasing amount of drug that can target cell. In contrast expression of COL11A1 gene was downregulated in 7 from 8 drug-resistant cell lines. It is possible that downregulation of this gene is rather general than drug specific response to cytostatics treatment.

High expression level of LOX in A2780PR1 cell lines seems to be related to expression of many COL genes in this cell line. LOX is lysine oxidase responsible for crosslinking of collagens and elastin [25]. Overexpression of this enzyme seems to be crucial to promote tumour growth and metastasis in many cancers, including lung cancer [26, 27], colorectal cancer [28], and breast cancer [29]. It is possible that expression of LOX in A2780PR1 cell line leads to its more invasive character. However, this requires further study.

Transforming growth of factor-beta-induced protein (TGFBI, also known as *β*ig-H3 and keratoepithelin) plays a role in a wide range of physiological and pathological conditions including tumorigenesis [30]. Depending on tumour microenvironment it can play as a tumour suppressor [31] or promoter [32]. Here we observe its increased expression in both Top-resistant cell lines. This suggests its role in resistance to this cytostatic drug. To our knowledge the role of TGFBI in Top resistance has not been described so far. TGFBI can bind to types I, II, and IV collagens and may play an important role in cell-collagen interactions. Thus its expression in Top-resistant cell line may result from its interaction with COL1A2 overexpressed in both Top-resistant cell lines.

Laminins are major proteins in the basal lamina. They influence cell differentiation, migration, and adhesion [33]. They also play a role in invasive behaviour of tumour cells. In investigated cell lines we observed different pattern of laminins expression. LAMB1 has been increased in both Pac-resistant cell lines. This suggests that it can be employed in resistance to this drug. The role of LAMB1 has been reported in malignant epithelial to mesenchymal transition, leading to more invasive phenotype [34]. Increased expression of laminins in drug-resistant breast cancer cell lines has also been reported [8]. In contrast LAMA1 and LAMA2 expression were decreased in A2780PR1 and A2780CR2 cell lines, respectively. LAMA1 downregulation is in contradiction to result of Işeri et al., who observed strong increase in LAMA1 expression in MCF-7 drug-resistant sublines [8]. Thus LAMA1 expression after cytostatic treatment may be cell line dependent. Changes in LAMA2 gene have not been reported so far in drug resistance cell lines.

We observed very high expression of EFEMP1 in A2780DR1 cell line. EFEMP1 is a member of the fibulin family of extracellular matrix glycoproteins. Its role in tumour suppression [35] as well as progression has been described [36]. It has been reported that EFEMP1 expression promotes angiogenesis and associates with lymph node metastasis, vascular invasion, and poor prognosis of cervical carcinoma [37]. In pancreatic adenocarcinoma EFEMP1 expression promoted tumour grow* in vivo* and rescued tumour cells from apoptosis induced by 5-fluorouracil, gemcitabine, and irinotecan [38]. We can suppose that overexpression of this gene in A2780DR1 cell line can be related to its antiapoptotic effect in response to Dox treatment.

Glypican (GPC), decortin, epiphycan, keratocan (KERA), lumican (LUM), and syndecan (SDC) are ECM proteoglycans. Some glypicans play a role in cell proliferation and survival. Expression of GPC6 has been observed in drug-resistant breast cancer cell lines [8]. In contrast to this study we observed strong downregulation of GPC6 but upregulation of GPC3 and GPC4 in some cell lines. GPC3 is frequently silent in ovarian cancer cell lines and seems to play as a tumour suppressor in ovarian [39] and lung cancer [40]. In contrast, it regulates cell proliferation in hepatocellular carcinoma—Huh7 Cell Line [41]. GPC3 and GPC4 have been downregulated in oxaliplatin-resistant ovarian carcinoma cell line A2780/C10 [42]. Taken together the role of glypicans in drug resistance is unclear.

KERA and LUM are members of the small leucine-rich proteoglycan (SLRP) family. KERA downregulation in Pac- and Cis-resistant cell lines is difficult to explain. To our knowledge relation of this gene to drug resistance has not been described so far. It can result from unspecific effect on these genes expression. Very high LUM downregulation in Pac-resistant cell line in the context of drug resistance is difficult to explain. LUM overexpression has been reported in Cis-resistant head and neck squamous cell carcinoma cell lines and in patients not responding to treatment with Cis-based combination chemotherapy [43]. Thus the role of LUM in Pac resistance requires further investigation. SDC2 is a transmembrane (type I) heparan sulfate proteoglycan. It is as an integral membrane protein and participates in cell proliferation, cell migration, and cell-matrix interactions. We observe overexpression of SDC2 in one of Pac-, Cis- and Dox-resistant cell lines. High expression of this gene has also been observed in MCF-7 cell lines resistant to Pac, Doc, and Dox [8]. Thus increased expression of SDC-2 can be related to drug-resistant phenotype of cancer cells.

Matrix gla protein (MGP) is a protein found in numerous body tissues. The encoded protein is found in the organic matrix of bone and cartilage and likely acts as an inhibitor of bone formation. The role of this protein in drug resistance has not been described so far. We observe its expression in four drug resistance cell lines with very high expression in Pac and Top resistance cell lines. These cell lines also express high level of laminin and collagen genes. It is possible that MGP can play a role in collagen and laminin metabolism.

Matrix metalloproteinase (MMP) is a family of zinc proteases involved in degradation of extracellular matrix in physiological and in disease condition, such as arthritis and metastasis. They are also responsible for cleaving number of bioactive molecules like cell surface receptors and play an important role in cell proliferation, differentiation, migration, angiogenesis, and apoptosis. In our study we observe overexpression of three MMPs: MMP1, MMP3, and MMP12. MMP1 is responsible for degradation of collagen types I, II, and III. In our study we observe overexpression of MMP1 in A2780PR1 and A2780TR2 cell lines. Both cell lines overexpress COL1A2. Thus overexpression of MMP1 in these cell lines can be related to COL1A2 overexpression and more invasive character of these cell lines. MMP1 overexpression has been reported in Dox-resistant breast cancer cell line [44]. MMP1 overexpression tended to be associated with a higher risk of progression in ovarian serous papillary carcinomas and is possible chemoresistance marker in this cancer [45]. Thus MMP1 can be responsible for more invasive phenotype of drug-resistant cancers. MMP3 is responsible for degradation of fibronectin, laminin, collagens III, IV, IX, and X, and cartilage proteoglycans. In A2780PR1 we observe very high expression level of MMP3 as well as LAMB1, LAMB3, and collagens: COL16A1, COL17A1, COL18A1, and COL21A1. We can suggest that MMP3 expression is related to LAMB1 and LAMB3 in this cell line. It can also be involved in degradation of COL16A1, COL17A1, COL18A1, and COL21A1 and more invasive behavior of these cell lines. Increased expression of MMP3 has also been reported in oxaliplatin-resistant ovarian carcinoma cell line A2780/C10 [42]. In head and neck squamous cell carcinoma high expression level of MMP3 was correlated with 5A/5A genotype and with the worst response to 5FU-cisplatin neoadjuvant chemotherapy [46]. We observe its overexpression in Pac-resistant ovarian cancer cell line. Thus MMP3 may be a potential marker of drug resistance. MMP12 is metalloproteinase with elastolytic activity. Its expression has been reported in oxaliplatin-resistant A2780/C10 ovarian cancer cell line [42]. In our study we observe its overexpression in A2780DR2 cell line resistant to Dox but not in Cis-resistant cell line. Different pattern of MMP10 expression has been observed. Overexpression was observed in A2780PR1 and A2780TR2 cell lines. However in A2780PR2, A2780CR1, and A2780DR1 we observe downregulation of this protein. MMP10 degrades proteoglycans and fibronectin. In A2780TR2 cell line MMP10 can be responsible for degradation of GPC3 and GPC4 leading to more invasive character of these cells. The role of MMP10 in maintenance and tumorigenicity of mouse lung cancer stem-like cells has been described [47]. In Cis-resistant A2780 ovarian cancer cell line increased expression of MMP10 has also been observed [48].

TIMP3 belongs to tissue inhibitor of metalloproteinases gene family which includes inhibitors of the matrix metalloproteinases, involved in degradation of the extracellular matrix (ECM). In our cell lines we observe strong overexpression of TIMP3 in A2780CR1 cell line and some increase in expression in A2780CR2 cell line, both resistant to Cis. However we do not observe increase of metalloproteinases expression in these cell lines. Our result is contradictory to results from drug-resistant breast cancer cell lines. In this study strong downregulation of TIMP3 was observed in Doc- and Dox-resistant cell line [8]. Downregulation of TIMP3 was also observed in ovarian tumors obtained after adjuvant chemotherapy [49]. In this context it is difficult to explain the role of TIMP3 in our drug-resistant cell lines.

In our study we have shown changes in expression of many genes encoding ECM and related proteins in eight drug-resistant ovarian cancer cell lines. The expression profiles presented here provide preliminary insight into differential expression of ECM and related genes in drug-resistant ovarian cancer cell lines. We did not observe any general response to cytostatic treatment. Changes in genes expression seem to be rather cytostatic dependent than general response to chemotherapy. However, the similarity between two cell lines resistant to the same drug is also not prominent. Significance of these gene expressions in drug resistance requires further analysis and should be confirmed on other ovarian cancer cell lines.

## Figures and Tables

**Table 1:** The gene list showing the fold-change in extracellular matrix related proteins in the drug-resistant sublines with respect to the parental A2780 cell line.

Gene symbol	RefSeQ transcript ID	Fold-change							
		C versus P1	C versus P2	C versus C1	C versus C2	C versus D1	C versus D2	C versus T1	C versus T2
ITGB8	NM_002214	NS	NS	NS	NS	NS	NS	**19.97**	**5.35**
COL1A2	NM_000089	**5.54**	NS	NS	NS	NS	NS	**56.50**	**66.36**
COL4A1	NM_001845	NS	**8.05**	NS	NS	NS	NS	NS	NS
COL11A1	NM_001854	**−29.34**	**−149.24**	**−106.43**	**−56.2398**	**−81.08**	NS	**−57.68**	**−24.29**
COL12A1	NM_004370	NS	NS	**23.92**	NS	**16.35**	NS	NS	NS
COL15A1	NM_001855	NS	NS	NS	NS	NS	NS	NS	**10.17**
COL16A1	NM_001856	**8.85**	NS	NS	NS	NS	NS	NS	NS
COL17A1	NM_000494	**16.79**	NS	NS	NS	NS	NS	NS	NS
COL18A1	NM_030582	**5.90**	NS	NS	NS	NS	NS	NS	NS
COL21A1	NM_030820	**55.13**	NS	NS	NS	NS	NS	NS	NS
LOX	NM_002317	**35.03**	NS	NS	NS	NS	NS	NS	NS
SPARC	NM_003118	NS	NS	NS	NS	NS	NS	NS	**17.71**
TGFBI	NM_000358	NS	NS	NS	NS	NS	NS	**27.94**	**17.48**
LAMA1	NM_005559	**−18.76**	NS	NS	NS	NS	NS	NS	NS
LAMA2	NM_000426	NS	NS	NS	**−33.4055**	NS	NS	NS	NS
LAMA4	NM_001105206	NS	NS	NS	NS	NS	NS	**9.52**	**8.92**
LAMB1	NM_002291	**15.89**	**93.16**	NS	NS	**8.65**	NS	NS	NS
LAMB3	NM_000228	**8.24**	NS	NS	NS	NS	NS	NS	NS
FN1	NM_002026	**−5.27**	**−5.049**	NS	NS	NS	NS	NS	NS
FLRT2	NM_013231	NS	NS	**6.37**	NS	NS	NS	NS	NS
FBN1	NM_000138	NS	NS	**−9.21**	**−13.3875**	NS	NS	NS	NS
EFEMP1	NM_001039348	NS	NS	NS	NS	**35.33**	NS	NS	NS
GPC3	NM_004484	NS	NS	NS	NS	NS	NS	NS	**23.71**
GPC4	NM_001448	NS	NS	NS	NS	NS	NS	NS	**19.80**
GPC6	NM_005708	**−33.30**	NS	NS	NS	NS	NS	**−16.01**	NS
DCN	NM_001920	Ns	**−10.52**	NS	NS	NS	NS	NS	NS
EPYC	NM_004950	NS	NS	NS	NS	**8.52**	**13.7955**	NS	NS
KERA	NM_007035	**−10.79**	**−7.94**	**−9.65**	**−6.08735**	NS	NS	NS	NS
LUM	NM_002345	NS	**−48.58**	NS	NS	NS	NS	NS	NS
HAPLN1	NM_001884	**−8.73**	NS	**10.65**	NS	NS	**9.29074**	**5.21**	**9.46**
SDC2	NM_002998	NS	**22.54**	**13.29**	NS	**19.13**	NS	NS	NS
KRT8	NM_002273	NS	NS	NS	NS	NS	NS	NS	**8.12**
MGP	NM_000900	**62.13**	NS	NS	**6.21346**	**9.13**	NS	**82.90**	**241.13**

NS: up- or downregulation between 5 and −5, indicative of changes in expressions that are not statistically significant.

**Table 2:** The gene list showing the fold-change in matrix metallopeptidases and related genes in the drug-resistant sublines with respect to the parental A2780 cell line.

Gene symbol	RefSeQ transcript ID	Fold-change							
		C versus P1	C versus P2	C versus C1	C versus C2	C versus D1	C versus D2	C versus T1	C versus T2
MMP1	NM_001145938	**11.65**	NS	NS	NS	NS	NS	NS	**10.63**
MMP3	NM_002422	**58.85**	NS	NS	NS	NS	NS	NS	NS
MMP10	NM_002425	**9.84**	**−17.77**	**−16.29**	NS	**−12.87**	NS	NS	**6.92**
MMP12	NM_002426	NS	NS	NS	NS	NS	**10.78**	NS	NS
ADAMTS3	NM_014243	**−7.95**	NS	NS	NS	NS	NS	NS	NS
ADAM22	NM_004194	NS	**8.05**	NS	NS	NS	NS	NS	NS
TIMP3	NM_000362	NS	NS	**50.82**	**6.24**	NS	NS	NS	NS

NS: up- or downregulation between 5 and −5, indicative of changes in expressions that are not statistically significant.
